# Silicosis, Sarcoidosis, and Silicosarcoidosis Are Overlapping Diagnoses and Difficult to Differentiate

**DOI:** 10.1002/ajim.70063

**Published:** 2026-02-15

**Authors:** Guilherme Ward Leite, Eduardo Kaiser Ururahy Nunes Fonseca, Rodrigo Caruso Chate, Lavinia Clara Del Roio, Sofia de Paiva Memento Machado, Jefferson Benedito de Freitas, Ronaldo Adib Kairalla, Mário Terra‐Filho, Ubiratan Paula Santos, Rafael Futoshi Mizutani

**Affiliations:** ^1^ Pulmonary Division, Instituto do Coracao (InCor), Hospital das Clinicas HCFMUSP, Faculdade de Medicina Universidade de Sao Paulo Sao Paulo Brazil; ^2^ Centro de diagnóstico por Imagem, Instituto do Coracao (InCor) Hospital das Clinicas HCFMUSP, Faculdade de Medicina Universidade de Sao Paulo Sao Paulo Brazil; ^3^ Hospital Israelita Albert Einstein São Paulo Brazil; ^4^ Faculdade de Medicina FMUSP Universidade de Sao Paulo Sao Paulo Brazil

**Keywords:** occupational lung disease, sarcoidosis, silica exposure, silicosarcoidosis, silicosis

## Abstract

We evaluated 12 workers with documented exposure to respirable crystalline silica who were referred to a tertiary care center due to clinical suspicion of silicosis, sarcoidosis, or silicosarcoidosis. Although silica exposure is a well‐established risk factor for silicosis and has been associated with autoimmune diseases, mycobacterial infections, and lung cancer, growing evidence also suggests a link with sarcoidosis, creating important diagnostic and therapeutic challenges. All patients underwent at least two clinical evaluations, high‐resolution computed tomography (HRCT), and pulmonary function tests over a minimum follow‐up of 12 months. Diagnostic classification was based on predefined criteria integrating occupational exposure history, longitudinal clinical course, HRCT patterns, pulmonary function changes, and histopathology when available; ten patients underwent transbronchial biopsy and six bronchoalveolar lavage. Three patients were classified as having silicosis, five as sarcoidosis, and four as silicosarcoidosis. Despite comparable exposure histories, the groups demonstrated distinct radiologic features, functional trajectories, and responses to therapy. Overlapping HRCT findings and the absence of standardized diagnostic criteria contributed to classification challenges, particularly in cases with mixed granulomatous and fibrotic patterns. In silica‐exposed workers, although silicosis remains the most frequent diagnosis, distinguishing it from sarcoidosis and silicosarcoidosis requires systematic longitudinal assessment. The integration of occupational history with serial clinical, radiologic, functional, and histopathologic evaluation enhances diagnostic accuracy and supports appropriate therapeutic decision‐making.

## 1 Introduction

Occupational exposure to respirable crystalline silica is associated not only with silicosis but also with pulmonary neoplasms [1, 2], tuberculosis and nontuberculous mycobacterial infections [3, 4, 5], autoimmune diseases [6], and chronic obstructive pulmonary disease (COPD) [7]. More recently, increasing evidence has suggested a potential association with sarcoidosis [8, 9]. The substantial radiologic overlap between silicosis and sarcoidosis complicates differential diagnosis in silica‐exposed workers. While sarcoidosis is a granulomatous disease and silicosis is characterized by fibrotic silicotic nodules, both conditions may present similar imaging findings; therefore, in many cases, differentiation relies on an integrated evaluation of high‐resolution computed tomography (HRCT), occupational exposure history, and, in selected cases, histopathological confirmation. Imaging reviews of pneumoconiosis and granulomatous lung diseases highlight potentially useful distinguishing features, including micronodule distribution patterns, the presence and type of lymph node calcifications, and the identification of progressive massive fibrosis [10]_._

Epidemiologic studies have long demonstrated an inverse association between smoking and sarcoidosis, with lower incidence among current and former smokers compared with never‐smokers [11]. However, this relationship may differ in individuals with substantial silica exposure. A large Swedish cohort of construction workers showed that high exposure to respirable crystalline silica was associated with an increased risk of sarcoidosis particularly among ever‐smokers, whereas the risk was not clearly elevated among non‐smokers [12]. These findings suggest a possible interaction between silica exposure and smoking—rather than a direct causal effect of tobacco alone—and should therefore be interpreted with caution. These findings, demonstrating an increased risk of sarcoidosis among individuals exposed to higher concentrations of respirable crystalline silica, support the biological plausibility of an interaction between environmental and occupational exposure and genetic susceptibility or epigenetic mechanisms [13, 14]. Several immunologic pathways implicated in the pathogenesis of sarcoidosis are also involved in silicosis, including activation of the NLRP3 inflammasome [15, 16] which has been shown to play a central role in silica‐induced inflammation and granuloma formation. However, disentangling the individual and interactive contributions of these risk factors remains challenging. Establishing such relationships would require large, long‐term prospective studies, which are difficult to conduct given the relative rarity of sarcoidosis. Multicenter collaborative studies may therefore represent a more feasible approach to further clarify these mechanisms [13, 14].

A recent study introduced the term silicosarcoidosis to describe cases exhibiting overlapping clinical, radiologic, and histopathologic features of both silicosis and sarcoidosis [17]. In this context, the present study characterizes the clinical, radiologic, functional, and pathological findings of 12 silica‐exposed workers and discusses practical considerations for the differential diagnosis of silicosis, sarcoidosis, and silicosarcoidosis.

This discussion is pertinent only in the context of well‐documented and sustained silica exposure accompanied by characteristic radiological abnormalities, circumstances under which the diagnosis of chronic silicosis is established. In such cases, there is no a priori indication for invasive diagnostic procedures to substantiate the diagnostic hypothesis, particularly given that silicosis is by far the most prevalent condition among individuals exposed to silica, as opposed to sarcoidosis.

## 2 Characteristics of Diseases

### 2.1 Silicosis

Silicosis is the most prevalent pneumoconiosis worldwide [18] still with the greatest illness and mortality burden, both in Brazil and globally [19, 20, 21]. It is characterized by persistent lung inflammation triggered by inhaled crystalline silica particles, leading to the formation of fibrotic nodules composed of a central collagen core surrounded by mononuclear inflammatory cells [19, 20, 21, 22, 23].

Pulmonary fibrosis is irreversible, and functional impairment, radiologic progression, and symptoms generally worsen according to cumulative inhalational burden, even after exposure cessation [3, 24]. Diagnosis is based on a compatible occupational exposure history, characteristic imaging findings, and exclusion of alternative diseases.

### 2.2 Sarcoidosis

Sarcoidosis has traditionally been considered a multisystem granulomatous disease of unknown etiology; however, this concept has been increasingly challenged by evidence implicating environmental and occupational exposures, including silica—as potential triggers [9, 25, 26]. The disease is characterized by non‐caseating granulomas that may involve mediastinal and hilar lymph nodes, lung parenchyma, or extrapulmonary organs such as the heart, eyes, central nervous system, skin, liver, and spleen [25]. Diagnosis requires integration of clinical, radiologic, and histopathologic findings, together with exclusion of alternative causes [27].

Sarcoidosis may spontaneously regress, remain stable, respond to immunosuppressive therapy, or progress. Epidemiologic data most recently support an association between silica exposure and increased sarcoidosis risk. A Swedish construction‐worker cohort demonstrated an 83% higher incidence among those with moderate to high exposure, with an even greater risk among current and former smokers [12]. Another Danish cohort likewise found that even relatively low cumulative silica exposure was associated with a significantly increased incidence of pulmonary sarcoidosis [28]. The clinical, radiologic, and occupational overlap between sarcoidosis and silicosis may both hinder definitive diagnosis and directly influence clinical management.

### 2.3 Silicosarcoidosis

Silicosarcoidosis has increasingly been recognized as an emerging diagnostic entity resulting from the interaction between genetic predisposition and silica exposure. Imaging features often overlap with both silicosis and sarcoidosis, and histopathology may demonstrate coexistence of silicotic nodules and epithelioid granulomas within pulmonary or lymph node tissue. Clinical presentation may include spontaneous or treatment‐related partial regression [17], although clear diagnostic criteria have not yet been established.

## 3 Methods

This observational study included 12 workers with a history of occupational silica exposure who were evaluated at the Occupational and Pulmonology Outpatient Clinic of the Division of Pulmonology, Instituto do Coracao (InCor), a tertiary care center in São Paulo, Brazil. Based on occupational history and initial imaging findings, silicosis was the preliminary diagnostic hypothesis in all cases; however, clinical, radiologic, and functional trajectory during follow‐up prompted further assessment to differentiate silicosis, sarcoidosis, or a combination of both.

To enhance diagnostic accuracy, all participants underwent at least two clinical evaluations, two high‐resolution computed tomography (HRCT) scans, and two pulmonary function tests (PFTs), with a minimum follow‐up duration of 12 months. Both retrospective and prospective data were analyzed, including occupational exposure history, clinical trajectory, radiologic progression, and pulmonary function changes. Transbronchial biopsy (TBB) was performed in 10 patients, and bronchoalveolar lavage (BAL) in six, using the aScope™ 4 Broncho Slim device.

Although TBB has a lower diagnostic yield than surgical lung biopsy for diffuse granulomatous and nodular lung diseases, it remains widely used because, when interpreted in conjunction with exposure history, radiologic findings, and BAL analysis, it frequently provides adequate tissue sampling for diagnostic purposes [29, 30]. This multimodal approach may reduce the need for more invasive procedures, such as thoracoscopic lung biopsy, particularly when silica exposure is well documented and granulomatous inflammation is identified. The presence of birefringent particles under polarized light was used as supportive evidence of mineral dust exposure, although differentiation between silica and silicate particles requires more precise complementary techniques such as scanning or transmission electron microscopy with X‐ray emission.

Histopathologic samples were reviewed by board‐certified pulmonary pathologists from the Division of Pathology, Hospital das Clinicas, University of São Paulo School of Medicine. After initial diagnostic reporting, all samples were subsequently reviewed and finalized by two experienced pulmonary pathologists. Imaging studies were independently evaluated by two thoracic radiologists and two pulmonologists with expertise in occupational lung diseases. Discrepancies were resolved by joint review by majority consensus.

The variables analyzed included: changes in dyspnea severity measured by the modified Medical Research Council (mMRC) scale; HRCT features such as micronodules distribution, conglomerate masses, hilar and mediastinal lymphadenopathy (with or without calcification), ground‐glass opacities, and fibrotic changes; pulmonary function variation defined as a ≥ 100 mL/year decrease in FVC and/or FEV₁; histopathologic findings; use of corticosteroids or immunosuppressive therapy; and the presence of extrapulmonary manifestations suggestive of sarcoidosis.

All data were anonymized prior to analysis. The institutional Research Ethics Committee of the Hospital das Clinicas approved the study and waived the requirement for individual informed consent due to its retrospective design (protocol SDC 5691/23/093).

## 4 Diagnostic Criteria

### 4.1 Silicosis

Silicosis was diagnosed based on a compatible occupational exposure history and characteristic high‐resolution computed tomography (HRCT) findings. Imaging typically demonstrates predominantly well‐defined centrilobular, peribronchovascular, and subpleural micronodules, occasionally exhibiting a mixed or random distribution, where a centrilobular and perilymphatic patterns may coexist. In more advanced or evolving diseases, large opacities may be observed, reflecting complicated silicosis or progressive massive fibrosis (PMF). The abnormalities predominantly involve the upper lobes. Hilar and mediastinal lymphadenopathy is common, frequently associated with lymph node calcifications. In cases of PMF, large opacities may exhibit central necrosis [31, 32, 33]. Pulmonary function was categorized as stable or declining between assessments. When performed, BAL typically demonstrated macrophage and neutrophil predominance, and transbronchial biopsy revealed silicotic nodules without epithelioid granulomas [20, 21, 24].

When lung tissue was available, polarized light microscopy was used to identify birefringent particles as supportive evidence of silica exposure. Absence of birefringent particles did not exclude clinically relevant exposure, particularly in settings involving fine or ultrafine silica particles (e.g., sandblasting or artificial‐stone cutting), where particle visualization may be limited and dense collagen may display similar optical properties [25].

### 4.2 Sarcoidosis

Sarcoidosis was characterized by HRCT findings with a predominance of perilymphatic micronodules, irregular interfaces, interlobular septal nodularity, and hilar or mediastinal lymphadenopathy, typically without calcifications. In advanced disease, pulmonary fibrosis with architectural distortion and ground‐glass opacities were observed. Conglomerate masses with associated bronchiectasis were occasionally present [27, 29, 30, 32]. We considered this presentation to represent sarcoidosis associated with silica exposure, rather than silicosarcoidosis, which is characterized by the concomitant features of both diseases. Pulmonary function could remain stable, improve, or worsen over time. When performed, BAL typically showed lymphocytosis > 15%, and biopsy demonstrated non‐necrotizing epithelioid granulomas [29, 30]. Clinical improvement or persistent radiologic or functional response to immunosuppressive therapy, or both, were also considered supportive of the diagnosis.

### 4.3 Silicosarcoidosis

Silicosarcoidosis was diagnosed when radiologic features of both silicosis and sarcoidosis were present—such as micronodules with mixed distribution patterns (centrilobular and perilymphatic), fibrotic masses, and lymphadenopathy with or without calcification [17]—combined with spirometric abnormalities and, when performed, BAL showed lymphocytosis > 15% and/or biopsy demonstrating both epithelioid granulomas and silicotic nodules [17].

This condition has been considered an emerging phenotype associated with silica exposure in genetically predisposed individuals [34, 35]. Although responses to immunosuppressive therapy are often less robust than in isolated sarcoidosis, treatment‐related radiologic or clinical improvement was incorporated into diagnostic classification. A more detailed description of all three conditions is provided in the Supporting Information S1.

## 5 Results

Twelve patients with suspected silicosis, sarcoidosis, or silicosarcoidosis were evaluated. The median follow‐up time was 6 years. Final diagnostic classification integrated occupational silica exposure with longitudinal clinical, radiologic, and functional trajectory, supplemented by histopathologic findings when available. Four patients were diagnosed with silicosarcoidosis, three with silicosis, and five with sarcoidosis. Baseline characteristics and exposure details are presented in Table 1. Median duration of silica exposure was 12 years (range 12–26) in the silicosis group, 12 years (range 1–34) in the sarcoidosis group and 11 years (range 7–40) in the silicosarcoidosis group. HRCT findings, symptom trajectory, pulmonary function changes, histologic features, and treatment are summarized in Tables 2 and 3.

**Table 1:** Occupational exposure data, age and smoking status of the patients.

Patients	Age (years)	Occupational activity	Smoking^a^
1	60	Stone polishing	No
2	44	Sandblasting	Yes
3	42	Tunnel drilling	Yes
4	60	Construction work	No
5	67	Marble work	Yes
6	60	Marble work	No
7	56	Marble work	No
8	41	Sandblasting	No
9	58	Ceramics machinery operator	No
10	49	Construction work	No
11	65	Marble work	Yes
12	43	Construction work	Yes

^a^ Yes includes both current and former smokers.

**Table 2:** Main imaging findings on chest HRCT scans.

Patient	Findings	Diagnosis
1	Calcified lymph nodes, ground‐glass opacities, centrilobular and perilymphatic micronodules, large opacities, bronchiectasis, and subpleural nodules/pseudoplaques	Silicosis
5	Calcified lymph nodes, centrilobular micronodules, bronchiectasis, and subpleural nodules/pseudoplaques	Silicosis
11	Calcified lymph nodes, mosaic attenuation and ground‐glass opacities, centrilobular and perilymphatic micronodules, large opacities, and subpleural nodules/pseudoplaques	Silicosis
2	Mosaic attenuation, perilymphatic micronodules, large opacities, and bronchiectasis	Sarcoidosis
3	Predominant perilymphatic micronodules, large opacities, and bronchiectasis	Sarcoidosis
7	Perilymphatic micronodules	Sarcoidosis
8	Enlarged lymph nodes without calcification, perilymphatic micronodules, large opacities, bronchiectasis, and subpleural nodules/pseudoplaques	Sarcoidosis
10	Calcified lymph nodes, perilymphatic micronodules, and large opacities	Sarcoidosis
4	Calcified lymph nodes, centrilobular and perilymphatic micronodules, large opacities, and subpleural nodules/pseudoplaques	Silicosarcoidosis
6	Calcified lymph nodes, ground‐glass opacities, centrilobular and perilymphatic micronodules, and large opacities	Silicosarcoidosis
9	Calcified lymph nodes, centrilobular and perilymphatic micronodules, large opacities, and subpleural nodules/pseudoplaques	Silicosarcoidosis
12	Calcified lymph nodes, predominant centrilobular and perilymphatic micronodules, large opacities, and subpleural nodules/pseudoplaques	Silicosarcoidosis

**Table 3:** Symptom progression, pulmonary function, imaging, pathology, and treatment.

Patient	Exposure time (years)	mMRC variation	PFT	PFT variation	HRCT variation	TBLB biopsy (NNG)	BAL (lymphocytosis ↑ )	Use of STC/IS:	Final diagnosis
1	12	0	OVD	2	0	No	No	No	Silicosis
5	12	1	RVD	2	1			No	Silicosis
11	26	1	MVD	0	2	No	No	Yes	Silicosis
2	11	0	OVD	2	0	Yes	No	No	Sarcoidosis
3	10	0	N	0	1	Yes	Yes	Yes	Sarcoidosis
7	34	0	OVD	1	1			No	Sarcoidosis
8	1	0	RVD	2	1	Yes	Yes	Yes	Sarcoidosis
10	31	0	RVD	0	1	Yes	Yes	Yes	Sarcoidosis
4	40	2	OVD	0	1	Yes	No	Yes	Silicosarcoidosis
6	9	0	OVD	1	1	No		Yes	Silicosarcoidosis
9	7	0	N	1	1	Yes		No	Silicosarcoidosis
12	11	2	MVD	0	2	Yes		Yes	Silicosarcoidosis

Abbreviations: 0, no change; 1, improvement; 2, worsening; BAL, bronchoalveolar lavage; HRCT, High‐resolution computed tomography; mMRC, Modified Medical Research Council dyspnea scale; MVD, Mixed ventilatory disorder; N, Normal; NNG, Non‐necrotizing epithelioid granuloma; OVD, Obstructive ventilatory disorder; PFT, Pulmonary function test; RVD, Restrictive ventilatory disorder; STC/IS, Steroid therapy/Immunosuppression; TBLB, Transbronchial lung biopsy; ↑, Lymphocytes > 15% of total BAL leukocytes.

### 5.1 Silicosis Group (Cases 1, 5, and 11)

Patients exhibited hallmark features of silicosis on chest CT, and in Case 11, histologic confirmation of only silicotic nodules. Functional impairment patterns varied: Case 1 demonstrated an obstructive ventilatory disorder, Case 5 a restrictive disorder, and Case 11 a mixed pattern. Functional decline occurred in Cases 1 and 5, whereas case 11 remained stable.

**Figure 1:**
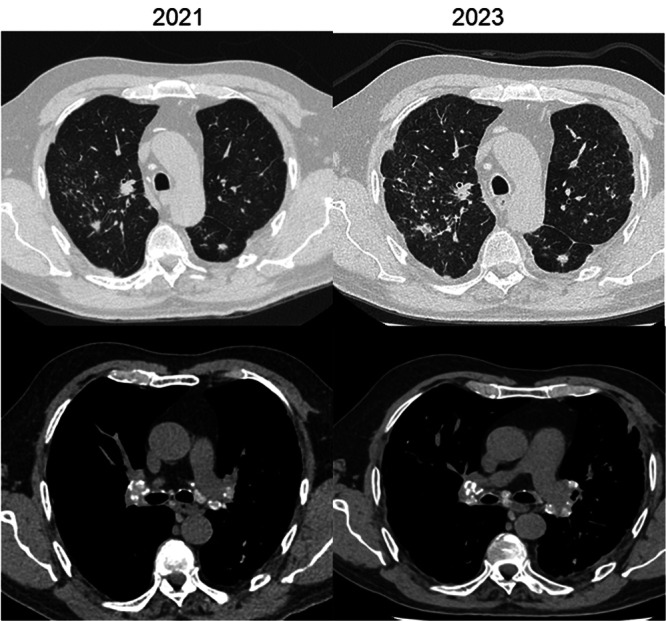
Silicosis (Cases 1, 5, and 11). Typical HRCT findings of *silicosis*, including upper‐lobe–predominant micronodules, pseudoplaques, and calcified hilar lymphadenopathy (“eggshell” pattern). Functional progression occurred despite radiological stability in two patients (Cases 1 and 5), emphasizing that physiologic decline may precede imaging deterioration.

Notably, Cases 1 and 5 experienced functional deterioration despite stable or improved HRCT findings, highlighting that physiological decline may precede radiologic progression. Case 5 showed symptomatic improvement following treatment of concomitant heart failure, emphasizing the need for comprehensive clinical evaluation, including other comorbidities. Case 11 received empirical immunosuppressive therapy early in follow‐up, which was discontinued after sarcoidosis was excluded (Figure 1).

### 5.2 Sarcoidosis Group (Cases 2, 3, 7, 8, and 10)

**Figure 2:**
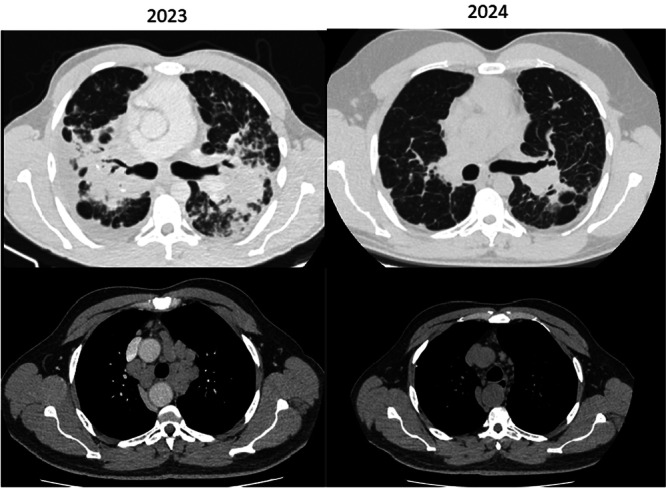
Sarcoidosis (Cases 2, 3, 7, 8, and 10). HRCT and functional follow‐up of patients diagnosed with *sarcoidosis*, illustrating perilymphatic micronodules, symmetrical mediastinal and hilar lymphadenopathy, and ground‐glass opacities. Most cases showed improvement after corticosteroid therapy, confirming the inflammatory nature of the disease.

This group demonstrated a more homogeneous pattern. Four of the five patients showed radiologic improvement with corticosteroid therapy. Non‐caseating granulomas and BAL lymphocytosis supported the diagnosis in three cases. Functional patterns varied, but all patients demonstrated stability or improvement. Symptoms remained mild and stable (mMRC 1) throughout follow‐up (Figure 2). No patient exhibited clinical signs of extrapulmonary involvement.

### 5.3 Silicosarcoidosis Group (Cases 4, 6, 9, and 12)

**Figure 3:**
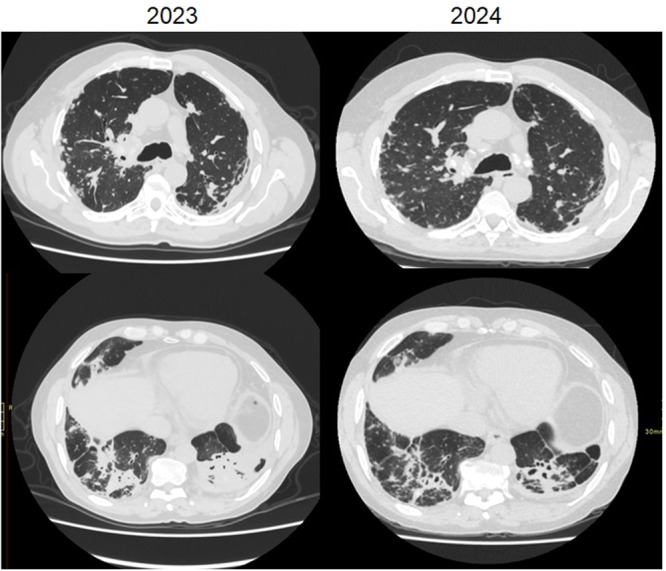
Silicosarcoidosis (Cases 4, 6, 9, and 12). Representative high‐resolution computed tomography (HRCT) images and clinical evolution of patients classified as *silicosarcoidosis*, showing mixed radiological features of both diseases—centrilobular and perilymphatic micronodules, fibrotic masses, and mediastinal lymph node calcifications. Three of four cases demonstrated radiological improvement after immunosuppressive therapy, particularly Cases 4 and 6, consistent with a partial inflammatory component.

This group comprised four patients. Radiological improvement following immunosuppressive therapy was observed in three cases (4, 6, and 9) (Figure 3). Histological confirmation of epithelioid granulomas was obtained in three patients. Pulmonary function improved in two cases (Cases 6 and 9), while the remaining two showed no significant functional variation (cases 4 and 12).

One of the characteristics that helps in distinguishing between the two forms is the presence of centrilobular micronodularity, combined with perilymphatic distribution and a higher frequency of calcified lymph nodes in silicosarcoidosis. In comparison, in cases of isolated sarcoidosis, perilymphatic distribution is by far the most common presentation and calcified lymph nodes are less frequent. These differences are indeed subtle, but they aid in diagnosis and treatment when combined with clinical, functional, and imaging evolution over time, as well with a histopathological analysis and bronchoalveolar lavage when possible.

## 6 Discussion

Differentiating chronic silicosis, sarcoidosis, and silicosarcoidosis is clinically and radiologically challenging, yet essential for the appropriate management of silica‐exposed individuals.

Silicosarcoidosis, an emerging and incompletely defined condition, showed partial response to immunosuppressive therapy in our patients. Three of four treated patients exhibited radiologic improvement, although this did not consistently translate into symptomatic or functional gains, suggesting that granulomatous inflammation alone may not fully account for clinical impairment.

Although several HRCT features may suggest overlap between silicosis and sarcoidosis, current evidence does not support the use of imaging alone as a reliable differentiating tool [19, 33]. Mixed radiologic patterns must therefore be interpreted alongside exposure history, longitudinal clinical assessment, pulmonary function testing, and bronchoscopy and histopathology when evaluated how necessary. This integrated approach remains critical for a more accurate diagnostic classification and for avoiding unnecessary or ineffective treatments.

Only one patient in the silicosarcoidosis group underwent the complete diagnostic evaluation, including both BAL and biopsy, and BAL did not demonstrate lymphocytosis. This emphasizes the limited diagnostic utility of BAL as an isolated criterion and reinforces the importance of relying on longitudinal, multimodal assessment rather than single biomarkers.

Silicosis remains an incurable condition, with management focused on secondary prevention, treatment of comorbidities, and surveillance for active or latent mycobacterial or fungal infections [36]. Corticosteroids are controversial and not routinely recommended due to limited evidence of benefit and increased infection risk, especially from tuberculosis [37]. Potential disease‐modifying therapies, including tetrandrine and tamoxifen, have yielded inconclusive results [38, 39, 40]. Additional challenges include limited understanding of disease mechanisms and low pharmaceutical investment in conditions of lower prevalence that disproportionately affect vulnerable worker populations. Lung transplantation may be considered in advanced cases, although there are several barriers, including restricted access to transplantation programs, limited availability of donor organs, insurance and coverage constraints, and anatomical or technical contraindications frequently seen in silicosis—such as extensive lymphonodal hilar calcifications and pleural adhesions, which may complicate the surgical procedure [41].

In contrast, sarcoidosis benefits from more well‐established treatment strategies, with favorable responses to corticosteroids and immunosuppressive agents such as methotrexate, azathioprine, and TNF‐alpha inhibitors, which act by attenuating the inflammation in most cases [33, 42]. This therapeutic contrast underscores the importance of accurate diagnostic distinction: under‐treatment of sarcoidosis may allow fibrosing progression, whereas inappropriate immunosuppression in silicosis may lead to infectious complications without clinical benefit.

For silicosarcoidosis, expert opinion supports short‐term immunosuppressive therapy with close monitoring for infectious complications [17]. Although recent studies [17] introduced the term silicosarcoidosis, its broad application to all silica‐exposed individuals with sarcoid‐like findings remains in debate. This reflects the substantial overlap in clinical, radiologic, and histopathologic patterns, along with possible differences in antigen‐driven immune mechanisms. Treatment strategies—primarily corticosteroids with additional immunosuppressants when needed—also overlap among these disorders. Collectively, these factors highlight the absence of a unified framework for defining and managing silica‐associated granulomatous lung diseases.

The term silicosarcoidosis should be used with caution, particularly given the potential for misclassification when occupational or environmental exposure histories are not rigorously assessed [35]. One report described a patient initially diagnosed with silicosis in whom lymphocyte proliferation testing demonstrated silica sensitization, suggesting silica‐induced immune activation rather than coexistence of two unrelated diseases [43]. Such observations underscore the importance of integrating exposure assessment and immunologic evaluation before applying this diagnostic label.

This study reinforces the need for a comprehensive diagnostic approach integrating occupational history, HRCT, pulmonary function testing, and BAL and histopathology when necessary to achieve a more accurate diagnosis.

Limitations include the small sample size (*n* = 12), which restricts generalizability—particularly for silicosarcoidosis; the observational design with mixed retrospective and prospective data; non‐standardized biopsy and BAL protocols across cases; and the absence of validated diagnostic criteria. Interpretation of radiologic and histologic findings, although performed by specialists, inherently involves some degree of subjectivity. Variable follow‐up durations and the lack of molecular or immunologic biomarkers further limit pathophysiological insights.

The differentiation between sarcoidosis and silicosarcoidosis was based on clinical, functional, and radiological evolution, as well as on histopathological analysis when available. Cases showing only epithelioid granulomas, accompanied by combinations of clinical, functional, or radiological improvement, and with absent or only minimal calcified lymphadenopathy, were classified as sarcoidosis.

This distinction is clinically relevant because therapeutic strategies differ across these conditions; however, the present case series is not designed to support treatment recommendations. While immunosuppressive therapy is an established option in selected cases of sarcoidosis, its role in silica‐associated granulomatous lung disease remains uncertain. In this study, treatment response was considered a supportive and exploratory element within a broader longitudinal and multimodal assessment, rather than a defining diagnostic criterion. Clinical response to immunosuppression in silica‐exposed patients is heterogeneous and may be influenced by disease stage, extent of irreversible fibrosis, and overlapping phenotypes, as reported in previous case reports and small series [34, 35]. Therefore, any consideration of immunosuppressive therapy in silicosarcoidosis should be interpreted with caution and cannot be generalized, underscoring the need for larger epidemiologic and multicenter studies before therapeutic strategies can be defined.

Nevertheless, our findings suggest that in silica‐exposed individuals with ambiguous radiologic features, detailed occupational assessment, bronchoscopy with BAL and tissue biopsy when necessary, and long‐term follow‐up remain fundamental for accurate diagnosis and optimal clinical management.

When evaluating a worker with a well‐documented history of silica exposure—considering the type of occupational activity, cumulative exposure burden, and latency period—together with radiological findings, particularly chest computed tomography, suggestive of chronic silicosis, the diagnosis of silicosis can be established without the need for invasive procedures.

Longitudinal monitoring of these patients, including their clinical, functional, and radiological evolution, has drawn our attention, as a subset of cases demonstrates a course that differs from what is typically observed in chronic silicosis. The present study aims to highlight the importance of regular follow‐up of individuals with current or past silica exposure, with careful assessment of their clinical, functional, and imaging trajectories, to ensure that the most appropriate management strategy is adopted for each patient.

Beyond diagnostic and therapeutic implications, differentiating silicosis, sarcoidosis, and silicosarcoidosis have broader occupational health and public health consequences. In countries such as Brazil, where exposure to respirable crystalline silica remains prevalent in mining, construction, and stone‐processing industries, achieving greater diagnostic accuracy directly influences not only clinical management but also the implementation of primary and secondary prevention strategies. Brazilian occupational health legislation assigns employers the responsibility for monitoring environmental risks and their potential health effects on workers, while public authorities are responsible for regulatory oversight and enforcement. This regulatory framework has been associated with underreporting occupational diseases. In the context of the present discussion on the potential role of silica exposure in the induction of sarcoidosis, clarifying the health implications of silica exposure may contribute to improved surveillance of exposed workers, similar to what has been established for autoimmune diseases such as rheumatoid arthritis, systemic sclerosis, and systemic lupus erythematosus, which have long‐standing evidence of association with silica exposure.

## 7 Conclusion

Differentiating silicosis, sarcoidosis, and silicosarcoidosis remains a significant clinical challenge due to substantial overlap in radiologic, functional, and histopathologic features among silica‐exposed individuals. Our findings highlight the necessity of integrating occupational history with longitudinal clinical assessment, pulmonary function testing, serial HRCT evaluation, and, when available, bronchoscopy with BAL and tissue biopsy. Long‐term follow‐up is essential to characterize disease trajectories, refine diagnostic classification, and guide therapeutic decisions tailored to individual patient response.

## Author Contributions

**Guilherme Ward Leite:** conceptualization, study design, data acquisition, data analysis, interpretation of results, and manuscript drafting. **Sofia de Paiva Memento Machado** and **Jefferson Benedito de Freitas:** data acquisition, clinical evaluation, patient follow‐up, and critical revision of the manuscript. **Eduardo Kaiser Ururahy Nunes Fonseca** and **Rodrigo Caruso Chate:** radiological analysis and interpretation of imaging studies, and manuscript revision. **Lavinia Clara Del Roio:** methodology support, data interpretation, and manuscript revision. **Ronaldo Adib Kairalla, Mário Terra‐Filho, Ubiratan Paula Santos**, and **Rafael Futoshi Mizutani:** supervision, interpretation of results, and critical revision of the manuscript for important intellectual content. All authors reviewed and approved the final version of the manuscript.

## Funding

The authors received no specific funding for this work.

## Ethics Statement

This study was approved by the Research Ethics Committee of Hospital das Clinicas, Faculdade de Medicina, Universidade de São Paulo, and was conducted in accordance with the Declaration of Helsinki. Due to the retrospective design and the use of anonymized data, the requirement for informed consent was waived.

## Supporting information


**Table 1:** Most typical findings on chest HRCT scans.

## Data Availability

The data that support the findings of this study are available from the corresponding author upon reasonable request.
