# Supplementary material for: Silicosis, Sarcoidosis, and Silicosarcoidosis Are Overlapping Diagnoses and Difficult to Differentiate
Source: Am J Ind Med. 2026 Feb 15;69(5):313–22. doi: 10.1002/ajim.70063 (PMC13070281; doi:10.1002/ajim.70063)
Supplement: Supplementary file 1 — Table 1: Most typical findings on chest HRCT scans. [file AJIM-69-313-s001.docx]

Supplementary File

**Table 1 :** Most typical findings on chest HRCT scans

| **Imaging findings** | **Silicosis** | **Sarcoidosis** | **Silicosarcoidosis** |
| --- | --- | --- | --- |
| Micronodule distribution | Centrilobular, or random, peribronchovascular and subpleural | Perilymphatic: subpleural, septal, and peribronchovascular | Mixed: centrilobular, perilymphatic, peribronchovascular, and occasionally random |
| Predominant location | Upper lobes, posterior regions | Upper and middle lobes, with bilateral symmetry | Upper and middle lobes, with variable pattern depending on the predominant component |
| Interlobular septal irregular thickening | Possible | Common | May be present, typically with an irregular nodular pattern |
| Pulmonary masses (PMF or conglomerates) | Common in advanced disease (PMF) | Possible in fibrotic stages (stage IV) | Present in mixed fibrotic forms; may mimic both patterns |
| Traction bronchiectasis | Secondary to fibrosis (less frequent) | Common in advanced stages | Common when significant coexisting fibrosis is present |
| Hilar and mediastinal Lymph node | Common, with calcifications, frequently with an eggshell pattern | Common, usually without calcification | Common, with or without calcifications (presence of both is suggestive) |
| Subpleural distribution | Micronodules may be observed, sometimes resulting in pleural pseudoplaques | Common | Frequently |
